# Internet access and utilisation of adolescents attending a national centre for paediatric rheumatology

**DOI:** 10.1186/1546-0096-12-S1-P301

**Published:** 2014-09-17

**Authors:** Derek Deely, Emma MacDermott, Orla Killeen

**Affiliations:** 1 National Centre for Paediatric Rheumatology, Our Lady's Children's Hospital Crumlin, Dublin 12, Ireland

## Introduction

With emerging interactive and communication technologies now available, the internet has become one of the top health information resources for adolescents (Stinson et al., 2010). Adolescents are typically the early adapters of new technologies, with in particular, the internet providing innovative opportunities for engaging youths. Traditional sources of health information are now becoming defunct and young people are increasingly going online for health related information.

## Objectives

To assess internet access and utilisation of adolescents.

## Methods

A convenience sample of 25 patients completed an online questionnaire. The questionnaire assessed the following: 1. Adolescents access and utilisation of the internet. 2. Adolescents utilisation of the internet to access health related information

## Results

From the 25 respondents 52% were female, 48% male. The mean age was 14.5 years (S/D: 11-18 years).

### 1. Access & utilisation of the internet

100% stated they have access to the internet on a daily basis with 85% using the internet 7 days per week. The reported time spent online ranged from 1 to 9 hours per day, mean: 2.9 hours. Table 1.

**Table 1:** Respondents had numerous ways to access the internet

Mobile phone	76%
**Personal laptop**	68%

**Tablet**	60%

**Game Console**	40%

**Home PC**	76%

**School Computer**	36%

Table 1. Respondents had numerous ways to access the internet:

88% of participants had one or more profiles on social networking sites. These included facebook (100%) and Twitter (92%).

### 2. Adolescents utilisation of the internet to access health related information.

65% stated they use the internet to look up health information. Of these, 85% researched their own medical condition/diagnosis followed by medications (35%), pain/coping with pain (21%), other medical conditions (21%), alcohol and medication (14%) and sexual health (7%).

When asked to list recent search items used to look up health information the following were listed: pain, explaining arthritis to others, drinking alcohol on methotrexate, hypermobility syndrome, lupus, methotrexate, medications and pregnancy, and TNF medications.

52% of respondents use the internet to communicate with peers who have a similar diagnosis. Of these, 100% use a facebook page linked with a community support group for adolescents and young people with rheumatic diseases.

## Conclusion

The internet has become an important tool for many people with health concerns; adolescents being no exception. Health professionals must know how to guide and advise adolescents in need of health related information to material that is both reputable and of a high standard while being age appropriate and appealing (Skinner et al., 2003).

## Disclosure of interest

None declared.

